# Gender differences in pharmacokinetics and pharmacodynamics of methadone substitution therapy

**DOI:** 10.3389/fphar.2015.00122

**Published:** 2015-06-09

**Authors:** Manuela Graziani, Robert Nisticò

**Affiliations:** ^1^Vittorio Erspamer School of Physiology and Pharmacology, Sapienza University of RomeRome, Italy; ^2^Drug Addiction and Clinical Pharmacology Unit, University Hospital Umberto I, Sapienza University of RomeRome, Italy; ^3^Department of Biology, University of Rome Tor VergataRome, Italy

**Keywords:** gender differences, pharmacokinetics, pharmacodynamics, methadone treatment, toxicology

## Abstract

Gender-related differences in the pharmacological effects of drug are an emerging topic. This review examines gender differences in both pharmacokinetic and pharmacodynamic aspects of methadone, a long-acting opioid agonist that is prescribed as a treatment for opioid dependence and the management of chronic pain.

**Method:** We performed a search in the Medline database from 1990 to 2014 in order to find published literature related to gender differences in pharmacokinetics (PK) and pharmacodynamics (PD) of methadone.

**Results:** None of the studies were carried out with the primary or secondary aim to identify any gender differences in the pharmacokinetic profile of methadone. Importantly; high inter-subjects variability in PK parameters was found also intra female population. The reported differences in volume of distribution could be ascribed to the physiological differences between men and women in body weight and composition, taking into account that the dose of methadone was established irrespective of body weight of patients (Peles and Adelson, 2006). On the other hand, the few studies present in literature found no gender difference in some direct pharmacodynamic parameters. Some reports have suggested that female gender is associated with an increased risk for long-QT-related cardiac arrhythmias in methadone maintenance subjects.

**Conclusion:** Even though it may be too simplistic to expect variability only in one parameter to explain inter-individual variation in methadone response, we believe that a better knowledge of gender-related differences might have significant implications for better outcomes in opioid dependence substitution therapy in women.

## Introduction

The most recent statistical data in Europe (EMCDDA, 2014) indicate that females represent roughly one in four drug users entering drug treatment and one in five deaths directly related to drug use. However, although drug abuse is actually more common among men than women, a progressively higher percentage of women in the world abuse both legal and/or illegal psychoactive drugs (Greenfield et al., 2003; Becker and Hu, 2008), making studies regarding gender differences in drug abuse extremely relevant. Gender differences are referred to all phases of natural history of drug abuse, i.e., initiation (Green et al., 2002; Becker and Hu, 2008; Roy et al., 2011; Shand et al., 2011), escalation (Hernandez-Avila et al., 2004), relapse, and treatment retention (Lynch et al., 2002; Greenfield et al., 2007; Niv and Hser, 2007; Kennedy et al., 2013), as well as to adverse effects (Gupta et al., 2007), psychiatric comorbidity (Edokpolo et al., 2010; Sara et al., 2013), and the class of drug of abuse (Simoni-Wastila et al., 2004; Tetrault et al., 2008). Possible factors that could contribute to gender differences are related to PK and PD variables.

Heroin abuse, despite the large increase in the 1990s, decreased in the last decade, in spite of the ascending trends in designer drugs consumers (Schifano et al., 2005), and behavioral addictions (Martinotti et al., 2014). Unfortunately, more recent data (NSDUH, 2010) indicate that in US the number of heroin initiates was significantly higher than the average annual number between 2002 and 2008. Recent data from the National Institute of Drug Abuse (NIDA, 2014) highlight the unexpected association of prescription pain reliever abuse and heroin, indicating the possible transition of experimenting with non-medical prescription pain relievers through heroin abuse. Data from 24 European countries also show an overall decrease in the number of heroin clients. The number of first-time heroin clients increased from 2005 to 2007 and then decreased in 2011 (EMCDDA, 2014). In line with data on population of both genders, the 2007 US National Survey on Drug Use and Health indicates that the rate of current heroin use decreased between 2006 and 2007 from 0.06 to 0.02% per cent among females aged 12 or older.

Currently methadone (M) is the most commonly prescribed replacement therapy for opioid dependence (only in the US 40% are female patients). In fact, recent data (EMCDDA, 2014) indicate that in Europe buprenorphine is prescribed to about 20% of the substitution treatment opioid users, whereas almost 734.000 opioid users received substitution treatment in Europe during 2012.

Although more than 15 years ago the FDA recommended women may be increasingly included in clinical trials (U.S. Food and Drug Administration, 1993), to date, gender difference in pharmacological response to opioids is scarcely investigated and only few clinical studies on opioid drugs (such as heroin, opioid pain relievers, and the opioids in dependence treatment M and buprenorphine) include gender as a factor. Increasing the knowledge of pharmacological aspects related to opioid substitution therapy drugs with relevance to female and male difference is essential for a better understanding of factors that influence opioid use among women, and might contribute to the identification and clinical use of gender-tailored therapy.

## Aim of the Work

The goal of this review is to supply a selective summary of the literature analyzing pharmacokinetic and pharmacodynamic aspects of M with specific focus on data obtained in women. We have also considered a limited number of studies including only male subjects, with the aim to hypothesize some gender differences, and also to underscore paucity of data.

## Methods

We reviewed the literature and searched for published articles related to gender differences in opioids pharmacokinetics (PK) and pharmacodynamics (PD). The MEDLINE database was examined from 1990 to 2014, using PubMed. Main key words used were: methadone, opioid, substitution therapy, pharmacology, PK, PD, sex/gender difference, individually, and variously paired.

## Methadone: PK and PD

Knowledge on PK and PD of M, in relation to different demographic characteristics (age, sex, pregnancy), pathophysiology of patients (renal impairment), and also drug interactions may contribute to the understanding of the well known individual variability in response to M treatment; this may in turn lead to an optimization of M therapy. Nevertheless and despite the fact that women are more susceptible to some of the adverse effects of M, such as the lengthening of the QT interval (Pearson and Woosley, 2005), the studies to investigate M treatment were carried out mainly on samples from the male population (Chopra et al., 2008).

### Pharmacokinetics

Available literature between 1990 and 2014 related to the PK of M with particular reference to any gender difference is listed in **Table 1**.

**Table 1:** Pharmacokinetics (PK) studies on methadone (M).

Reference	Subjects N ( female)	Subjects category	Dosage regimen	Dose range (mg)	Biologic fluid of determination	Sex difference (female)
Bart et al. (2014)	206 (39)	I	Multiple dosing	35–120	Plasma	Sex data not published separately
Shiran et al. (2009)	88 (24)	I	Multiple dosing	15–130	Plasma	= (R)-, (S)- and (R)-(S)-M plasma C M clearance
Fredheim et al. (2007)	12 (42)	II	Multiple dosing	20–85	Plasma	Sex data on M and EDDP plasma C not published separately
Hanna et al. (2005)	6 (1)	I	Multiple dosing (no dose change greater than 15 mg during the last 6 months)	20-170	Plasma	Sex data not published separately
Foster et al. (2004)	59 (39)	I	Multiple dosing (no dose change for at least 2 months)	7.5–150	Plasma	>Vd ^∗∗∗∗^
Preston et al. (2003)	27 (3)	I	Multiple dosing (no dose change for at least 1 week)	35–80	Urine	>EDDP C
Boulton et al. (2001)	8 (100)	III	Single dose	0.2 mg/Kg	Plasma and urine	High inter-subjects variability in PK and PD parameters
Wolff et al. (2000)	35 (38)	I	Single dose	5–80	Plasma	Difference in Vd not specified; =M clearance
Foster et al. (2000)	18 (12)	I	Multiple dosing (no dose change for at least 2 months)	7.5–130	Plasma	= unbound fractions for (R)-M; = a1-acid glycoprotein C; = plasma C-time profiles for either (R)- or (S)-M;
Rostami-Hodjegan et al. (1999)	17 (6) 19 (13)	II I	Single dose Multiple dosing (Days 6–37)	5–80 5–80	Plasma	=Vd =Clearance
Preston et al. (2003)	27 (3)	I	Multiple dosing (no dose change for at least 1 week)	35–80	Urine	>Creatinine-corrected EDDP C >non-significantly higher M C =EDDP/M ratio
Eap et al. (1996)	22 (4.4)	I	Multiple dosing (R)-M and subsequently (R, S)-M	120–160	Plasma	Sex data not published separately
de Vos et al. (1995)	20	II	Multiple dosing	10–225	Plasma	<M clearance

*I = Methadone maintenance treatment, II = opiate users, III = drug free subjects, III = cancer patients.

** = Concentration.

*** = 2-ethylidene-3,3-diphenylpyrrolidine.

**** = Central volume of distribution (Vd).

#### Absorption and Distribution

As noted, M is characterized by high lipid solubility and protein binding (Foster et al., 2000; Ferrari et al., 2004; Vazquez et al., 2006). A study conducted to investigate the steady-state PK of (R)- and (S)-methadone in an M maintenance population (*N* = 18, 39% women) showed no gender-related effect on plasma concentration-time profiles after a multiple dosing regimen for either (R)-methadone or (S)-methadone, neither on α1-acid glycoprotein plasma concentration. Similarly, no effect of gender difference on initial Volume of distribution (Vd) in a sample of 35 opiate users (M single dose: *N* = 23; 26 % women; M multiple doses: *N* = 29; 45 % women was found (Rostami-Hodjegan et al., 1999); however, in this study, since M dose was in a range of 5–80 mg and plasma concentrations were assessed and normalized to the dose of 70 mg using a population-based pharmacokinetic approach, gender effect on dose-concentration relationship could not be evaluated. When higher values of central Vd were found in female users (Rostami-Hodjegan et al., 1999), this parameter was directly related to sex differences in body weight. Accordingly, Wolff et al. (1997) in a sample of 21 males e 14 females reported that the Vd of M was affected by weight, but not by gender. On the contrary, Foster et al. (2004), identified gender and plasma a1-acid glycoprotein concentration as significant determinants of apparent Vd of the central compartment (Vc/F). These data are consistent with those observed by Rostami-Hodjegan et al. (1999), which report that gender and weight (factors affecting body composition) were the two main covariates accounting for 33% of the inter-individual variability in M Vd at the steady-state.

#### Metabolism

M biotransformation is largely mediated by CYP3A4 and CYP2D6 (Eap et al., 2002), although CYP2B6 (Dennis et al., 2014), CYP2C9, and CYP2C19 also appear to be involved, but to a much lesser degree (Foster et al., 1999).

##### CYP3A4

CYP3A4 is responsible for M biotransformation in the major metabolite 2-ethylidene-1,5-dimethyl-3,3-diphenylpyrrolidine (EDDP; Eap et al., 2002). Studies (Charlier et al., 2001; Eap et al., 2002) aimed to identify the proper daily dose found a poor correlation between M doses and M serum concentrations. In another study (Boulton et al., 2001), performed on eight healthy drug-free women, the authors showed a correlation between urinary cortisol, area under the curve, and elimination of EDDP (a metabolite of M). Recently, Shiran et al. (2009) in a study conducted in a sample of 88 subjects (30 females) found that CYP3A4 activity has a modest influence on M disposition.

The discrepancy between these results could be affected by several factors: conditions of concentration at steady state vs. single dose, patients in methadone maintenance therapy (MMT) vs. volunteers not taking chronic opiates.

To date inter-individual variations in the expression of CYP3A4 are thought to be the main factor responsible for inter-individual variability in M response (Li et al., 2008): indeed, genetic polymorphism is considered the cause of high variability of M blood concentrations for a given dose.

Moreover, CYP3A4 seems to be the main isoenzyme of the CYP family showing significant sex differences, since its expression in women is 1.5–2 times higher than in men (Wolbold et al., 2003): the underlying mechanism is presumably type transcriptional, linked to the mRNA. Notably, CYP3A4 expression in the liver is controlled also by dynamic interactions with transcription factors such as expression of the ABCB1 (multidrug resistance 1 [MDR1]) gene encoding the P-glycoprotein (P-gp) eﬄux transporter (Schuetz et al., 2000). Since hepatic P-gp protein expression is twofold lower in women than in men (Schuetz et al., 1995) this could increase the intra-hepatic concentration of M and in turn its more extensive metabolism. In this regard, pharmacogenetic studies indicate that it is important to stratify by gender when examining genotype/phenotype associations, especially for genes, such as CYP3A4, which are differently expressed between genders (Lamba et al., 2003, 2006).

##### CYP2D6

CYP2D6 contributes only to a small extent to the formation of EDDP (Eap et al., 2002). It exhibits a high polymorphism: to date, over 75 different alleles have been identified (Borobia et al., 2009), differently expressed among ethnic groups (Xie et al., 2001).

Clinical data regarding gender differences in the activity of CYP2D6 are controversial. Some authors found no differences among males and females, including poor metabolizers subjects (Kashuba et al., 1998; Tamminga et al., 2001). Conversely, Labbé et al. (2000) found in a sample of extensive metabolizers subjects, that the metabolism of CYP2D6 substrate dextromethorphan, is higher in women than in men, as confirmed by Hägg et al. (2001).

Methadone maintenance therapy patients Shiran et al. (2003) observed a lower activity of CYP2D6 (rated metabolic ratio of dextromethorphan) in women; the results of this study revealed the presence of a substantial genotype/phenotype discrepancy in MMT patients, consistent with inhibition of CYP2D6. Authors speculate that the chronic intake of M might induce inhibition of CYP2D6, similarly, to the one observed in *in vitro* conditions (Wu et al., 1993). This hypothesis could explain, according to the authors, that the difference in activity observed in both genders may result from possible differences between themselves in the plasma concentration of M administered at the same dose. In any case, in a more recent study involving 88 subjects (including 30 women), Shiran et al. (2009) evaluated the possible influences of the activity of CYP2D6, 1A2, and 3A4 on the availability of M, reporting that gender did not have a significant influence, along with other covariates such as CYP activity, weight, and age, neither on the plasma concentration of M nor on its clearance.

#### Excretion

M undertakes hepatic metabolism and renal excretion (Garrido and Trocóniz, 1999). With respect to its hepatic elimination, M has a restricted clearance or a low extraction ratio, while, as a consequence of its chemical properties, urine pH has been shown to influence the excretion of M (Inturrisi et al., 1987). To our knowledge, there are no studies that have shown gender differences in the excretion of M; however, it is useful to point out that recently Poggio et al. (2009) observed that females had slightly but significantly higher glomerular filtration rate (measured by 125I-iothalamate clearances) than males after adjustment for body surface area, but there were no differences due to race. Instead, Berg (2006) found no difference in glomerular filtration rate by clearance of para-aminohippuric acid (PAH) between males and females, in a total of 122 potential kidney donors (62 females).

To summarize, none of the studies was carried out with the primary or secondary aim to identify any gender differences in the pharmacokinetic profile of M. Importantly, high inter-subjects variability in PK parameters was found also intra female population (Boulton et al., 2001) Anyway, when women are included in clinical studies and sex data were analyzed separately some significant differences were detected.

The physiological differences between men and women in body weight and composition (per cent of fat, plasma volume, and organ blood flow) could be evoked to explain difference in Vd, taking into account dose M is established irrespective of body weight of patients (Peles and Adelson, 2006). It can be hypothesized that, in turn, changes in Vd may in turn affect both the levels of plasma concentration and the half-life, with consequences on the effect of drugs in terms of onset of action and duration of effects.

### Pharmacodynamics

Clinical studies on M PK/PD relationship are listed in **Table 2**. None of these studies has been conducted to primarily identify a possible difference in M PD parameters in women compared to men; in most of the studies gender data are not analyzed separately. In a study conducted by Hiltunen et al. (1999), no influence of gender was found on the interaction between l-M plasma concentrations and subjective opiate withdrawal scale (SOWS) of “negative” (irritability, disorientation, remorse, sadness, and low psychomotor speed), and “positive” effects (feelings of alertness, calmness, patience, relaxation, cheerfulness, carefreeness, and clear-thinking). Peles and Adelson (2006) comparing gender outcome among 470 MMT patients (28% women) found no gender differences in treatment retention, stop in opiate abuse, and decrease in cocaine abuse. The average M dose was almost the same between men and women (131.1 ± 54.8; 132.3 ± 49.5 mg).

**Table 2:** Methadone (M) PK/PD relationship studies.

Reference	Subjects N (% female)	Subjects Category^∗^	Dosage regimen	Dose range (mg)	PD effect	Findings/ Comments
Hanna et al. (2005)	6 (16.6)	I	Repeated dose	20–170 mg/die	Antinociception, mood disturbance, respiration rate, pupil size, withdrawal.	Highly significant differences in the between- to within-subject variability. Small subjects number
Maremmani et al. (2005)	83 (24)	I	Repeated dose	10–600 mg/die	QTc prolongation	No correlation between QTc values and M doses; no gender differences in QTc values
Pearson and Woosley (2005)	58 (59)	I	Repeated dose	29–1680 mg/die	QTc prolongation and/or TdP	Female gender as risk factor; QT prolongation at therapeutic dose
Pérez de Los Cobos et al. (2005)	165 (23)	I	Repeated dose	12–150 mg/die	Patient M therapy satisfation	Female gender more satisfied
Fisher et al. (2004)	6 (50)	II	Repeated dose	1-4 X 1-25 mg/die	Antinociception	Patients’ heterogeneous condition; data not analyzed by gender; small subjects number
Mitchell et al. (2004)	55 (38)	I	Repeated dose	7.5–300 mg/die	Mood disturbance, respiration rate, pupil size, heart rate, blood pressure, withdrawal.	Gender data not published separately; M AUC^∗∗^ not related to PD responses, except for mood disturbance and heart rate
Dyer et al. (2001)	18 (39)	I	Repeated dose	7.5–130 mg/die	Mood disturbance	Gender data not published separately; M plasma concentration inversely related to mood disturbance
Boulton et al. (2001)	8 (100)	III	Single dose	0.2 mg/Kg	Pupil diameter	Large inter-subject variability
Dyer et al. (1999)	18 (39)	I	Repeated dose	7.5–130 mg/die	Withdrawal severity, Pain threshold, Pupil diameter, Respiratory rate	No differences between holders and non-holders with respect to gender
Gourevitch et al. (1999)	18 (56)	I	Repeated dose	30–100 mg/die	Withdrawal	Gender data not published separately; no correlation between self-reported symptoms of withdrawal and trough plasma M levels.
Hiltunen et al. (1999)	50 (24)	I	Repeated dose	35–140 mg/die	Objective and subjective withdrawal scale in relation to plasma M C^∗∗∗^	No gender differences on the interaction between l-M plasma concentrations and “negative” as well as of ”positive” M effects
Inturrisi et al. (1990)	15 (27)	II	i.v. infusion (180–270 min)	20–25 mg/hour (loading dose)	Relationship between plasma M C and PD effects (analgesia and sedation)	Sex data not published separately

* I = Methadone maintenance treatment, II = Patients with cancer-related pain, III = healthy subjects.

** = Area under the curve.

*** = Concentration.

On the contrary in another study (Pérez de Los Cobos et al., 2005) conducted on 169 patients (33 % women) to evaluate satisfaction with treatment (dose adjustment, participation in dosage regulation) it was found that female patients had more positive opinions and a better satisfaction than males about M as a medication. Accordingly, some authors (Green, 2006; Greenfield et al., 2007) observed that males were more expected to drop out of outpatient drug-free programs, whereas females were more likely to be considered in the low-retention group for outpatient M treatment (Simpson et al., 1997). In a more recent study aimed to ascertain gender and M maintenance dose influence on naloxone response in volunteers, a significant difference in mean M doses was observed between males and females (when body weight was considered; Chopra et al., 2008). These results indicate that women on lower M maintenance doses might be very sensitive to the dysphoric effects of naloxone-induced opiate withdrawal.

It should, however, be noted that no pharmacodynamic parameters were directly analyzed. Some studies have shown a difference in μ-opioid binding between males and females. Smith et al. (1998), using positron emission tomography (PET), observed a higher μ-opioid binding in women in a number of cortical and subcortical areas. Furthermore, gonadal steroid hormones such as estrogenic states can influence μ-opioid receptor density (related to opioid relative efficacy) and signal transduction (Smith et al., 1998). More recently Smith et al. (2006) have demonstrated that a high-estrogen state is related to area-specific increases in μ-opioid receptor availability, while during the low estrogenic conditions significant reduction in endogenous opioid tone was observed at the level of thalamus, nucleus accumbens, and amygdala. Age is another factor that can contribute to μ-receptor occupancy. In fact, in postmenopausal women the *in vivo* μ-opioid binding decreases to levels below those of men (Zubieta et al., 1999).

Some studies (Drici and Clément, 2001; Pearson and Woosley, 2005), although not equivocally (Maremmani et al., 2005), have suggested that female gender is associated with higher risk for long-QT (LQT)-dependent cardiac arrhythmias in MMT subjects. Reports to the FDA spontaneous reporting system (however, considered an underestimation of the total number of events) indicate that in a total of 5503 reports of adverse events associated with M, 1.07 % noted the occurrence of QT prolongation and TdP, and that 59% were females. Importantly, the dosages for 29% of the cases were within the recommended range for M maintenance treatment (60–100 mg/day), indicating that pharmacodynamic variables, not pharmacokinetic, might explain this cardiac effect.

Among the possible factors that can contribute to M cardiac toxicity, there may be physiological differences in cardiac conduction between genders: the baseline QTc of the post puberty female is longer than that of males (Abi-Gerges et al., 2004), suggesting that sex steroid hormones are implicated in modulating cardiac repolarization; drugs like M blocking cardiac voltage gated potassium channels, particularly the rapid component (IKr) of the delayed rectifier potassium current (IK), may induce a prolongation of the QT interval (Drici and Clément, 2001). In contrast, in a study (Maremmani et al., 2005) conducted to assess the incidence of abnormal QTc interval values in a population of subjects (20 females, 63 males) on a long-term MMT (60–100 mg/die), gender differences in the recorded QTc values were found not statistically significant. It should be noted, however, that the number of women enrolled in the sample was not very high, as it has not been clarified whether the average M dose was lower in women than in men.

In summary, considering the above mentioned inter women variability, the few studies present in literature found no gender difference in some direct pharmacodynamic parameters (withdrawal severity, pain threshold, pupil diameter, and respiratory rate, as well as in the interaction between l-M plasma concentration and its “negative”/“positive” effects. However, there is no consensus in the literature about gender differences in treatment satisfaction and retention in M substitution therapy. On the other hand, there is clear evidence of women’s vulnerability to M cardiac adverse effects that may be related to gender-related physiological differences in cardiac conduction.

## Conclusion

To date, data available on M PK and PD focuses primarily on male subjects. Little is known about women or how the genders compare. We hope that future studies will determine the exact contribution of the gender factor to the inter-individual variation of the pharmacology of M in patients. Even though it may be too simplistic to expect variability only in one parameter to explain inter-individual variation in M response, we believe that a better understanding of gender-related differences might have important implications for a better outcome in opioid dependence substitution therapy in women.

## Conflict of Interest Statement

The associate editor, Cesare Mancuso declares that, despite having collaborated with the author Robert Nisticò, the review process was handled objectively. The authors declare that the research was conducted in the absence of any commercial or financial relationships that could be construed as a potential conflict of interest.
